# Treatment of malignant effusion by oncolytic virotherapy in an experimental subcutaneous xenograft model of lung cancer

**DOI:** 10.1186/1479-5876-11-106

**Published:** 2013-05-01

**Authors:** Stephanie Weibel, Elisabeth Hofmann, Thomas Christian Basse-Luesebrink, Ulrike Donat, Carolin Seubert, Marion Adelfinger, Prisca Gnamlin, Christina Kober, Alexa Frentzen, Ivaylo Gentschev, Peter Michael Jakob, Aladar A Szalay

**Affiliations:** 1 Department of Biochemistry, Biocenter, University of Wuerzburg, Wuerzburg D-97074, Germany; 2 Department of Experimental Physics 5, University of Wuerzburg, Wuerzburg D-97074, Germany; 3 Research Center for Magnetic Resonance Bavaria e.V., Wuerzburg D-97074, Germany; 4 Rudolf Virchow Center, Research Center for Experimental Biomedicine, University of Wuerzburg, Wuerzburg D-97078, Germany; 5 Institute for Molecular Infection Biology, University of Wuerzburg, Wuerzburg D-97078, Germany; 6 Department of Radiation Medicine and Applied Sciences, UC San Diego Health System, La Jolla, CA 92093, USA; 7 Genelux Corporation, San Diego Science Center, 3030 Bunker Hill Street, Suite 310, San Diego, California 92109, USA

**Keywords:** Oncolytic virotherapy, Malignant effusion, Lung cancer, VEGF

## Abstract

**Background:**

Malignant pleural effusion (MPE) is associated with advanced stages of lung cancer and is mainly dependent on invasion of the pleura and expression of vascular endothelial growth factor (VEGF) by cancer cells. As MPE indicates an incurable disease with limited palliative treatment options and poor outcome, there is an urgent need for new and efficient treatment options.

**Methods:**

In this study, we used subcutaneously generated PC14PE6 lung adenocarcinoma xenografts in athymic mice that developed subcutaneous malignant effusions (ME) which mimic pleural effusions of the orthotopic model. Using this approach monitoring of therapeutic intervention was facilitated by direct observation of subcutaneous ME formation without the need of sacrificing mice or special imaging equipment as in case of MPE. Further, we tested oncolytic virotherapy using Vaccinia virus as a novel treatment modality against ME in this subcutaneous PC14PE6 xenograft model of advanced lung adenocarcinoma.

**Results:**

We demonstrated significant therapeutic efficacy of Vaccinia virus treatment of both advanced lung adenocarcinoma and tumor-associated ME. We attribute the efficacy to the virus-mediated reduction of tumor cell-derived VEGF levels in tumors, decreased invasion of tumor cells into the peritumoral tissue, and to viral infection of the blood vessel-invading tumor cells. Moreover, we showed that the use of oncolytic Vaccinia virus encoding for a single-chain antibody (scAb) against VEGF (GLAF-1) significantly enhanced mono-therapy of oncolytic treatment.

**Conclusions:**

Here, we demonstrate for the first time that oncolytic virotherapy using tumor-specific Vaccinia virus represents a novel and promising treatment modality for therapy of ME associated with advanced lung cancer.

## Background

More than 226,000 new cases of lung cancer are projected to occur in 2012 in the United States, and its high mortality rate makes it the leading cause of cancer-related death [1]. Malignant pleural effusion (MPE) is a common complication of patients with highly symptomatic and advanced-stages of lung cancer. In the clinic, these patients are difficult to manage and have, in general, a short life expectancy of 4–9 month after diagnosis [2]. In addition to lung cancer, MPE is a serious complication associated with different other tumor types including breast cancer and lymphomas affecting altogether 175,000 patients in the United States each year [3]. Today, only palliative therapies such as thoracentesis and chemical pleurodesis are available in the clinic [4]. Therefore, novel approaches for the therapy of MPE are needed.

Despite being a frequent and serious complication in cancer patients, the underlying mechanism of MPE formation is not fully understood. In all cases, fluid accumulates in the pleura as a result of increased pleural fluid formation and/or reduced drainage. However, increased local fluid formation is the principal underlying abnormality in the genesis of most exudative effusions, even if obstruction of fluid drainage co-exists [5]. MPE formation is considered to develop on the basis of a locally disturbed tumor-microenvironment, with leakiness of local endothelial cell layers and pleural invasion by tumor cells [6,7]. In various studies, vascular endothelial growth factor (VEGF) has been identified as a key mediator contributing to the formation of malignant effusions (ME) in solid tumors by modulation of the tumor vasculature [6,8]. Strategies to antagonize the VEGF activity at various target points of the corresponding signaling pathway have shown success *in vitro* and in animal models of ME and ascites [5,9-13].

Oncolytic virotherapy of tumors is an up-coming, promising therapeutic modality of cancer therapy based on the lytic destruction of solid tumors mediated by infection of the malignant tissue by tumor-specific viruses [14-17]. A multitude of different virus strains with oncolytic potential have been described and promising pre-clinical data as well as clinical trial reports from oncolytic virotherapy are available [18-20]. Recently, Zhang et al. [21,22] have introduced the attenuated recombinant Vaccinia virus (rVACV) GLV-1h68 which was used as an oncolytic agent in several pre-clinical tumor models [23-27] and is currently applied in clinical trials (http://www.clinicaltrials.gov; references NCT00794131 and NCT01443260).

In the present study, we showed that the oncolytic VACV GLV-1h68 and its derivative GLV-1h108 [28], which encodes for a single chain antibody (scAb) against VEGF revealed significant tumor growth control and prevented formation of ME in a subcutaneous advanced-stage lung adenocarcinoma model. In addition, we showed for the first time that oncolytic virotherapy led to a reduction of tumor cell-derived VEGF-levels, to decreased invasion of tumor cells into the peritumoral tissue, and to viral infection of blood vessel-invading tumor cells, thereby preventing formation of ME. These data support the use of oncolytic virotherapy against both the primary tumor as well as tumor-associated effusions.

## Methods

### Cell lines

PC14PE6-RFP human lung adenocarcinoma cells were stably transduced with the full-length *dsRed2* cDNA as described by Kienast el al. [29] and kindly provided to us by F. Winkler (University of Heidelberg, Neurooncology, Heidelberg, Germany) in 2008. The PC14PE6-RFP cells used in this study were authenticated by the Leibniz-Institut DSMZ (Deutsche Sammlung von Mikroorganismen und Zellkulturen GmbH, Braunschweig, Germany) by STR profiling to be identical with the parental cell line PC14 (Riken, Japan) in 2012. PC14PE6-RFP cells were cultured in DMEM supplemented with 1x MEM non-essential amino acids, 2 mM GlutaMAX, 10% FBS, 100 Units/ml penicillin, and 100 μg/ml streptomycin. Cells were maintained at 37°C and 5% CO_2_.

### Virus strains

Construction of the attenuated Vaccinia virus strains GLV-1h68 and GLV-1h108 was described previously by Zhang et al. [21] and Frentzen et al. [28], respectively. Briefly, three expression cassettes (encoding for *Renilla* luciferase-GFP fusion protein, β-galactosidase and β-glucuronidase) were recombined into the *F14.5L*, *J2R* and *A56R* loci, respectively, of the LIVP strain virus genome. In case of GLV-1h108 the *glaf-1* coding sequence was recombined into the *J2R* locus of the parental GLV-1h68 virus strain. Viruses were propagated in CV-1 cells and purified through sucrose gradients.

### Tumor inoculation and administration of the virus

All animal experiments were carried out in accordance with protocols approved by the Institutional Animal Care and Use Committee (IACUC) of Explora Biolabs (San Diego, USA, protocol number EB11-025) or the government of Unterfranken (Würzburg, Germany, protocol number AZ 55.2-2531.01-17/08).

Six-week-old female athymic nude *Foxn1*^*nu*^ mice were obtained from Harlan Laboratories (Netherlands and Indianapolis). PC14PE6-RFP tumor cells (4 × 10^5^/100 μl PBS) were subcutaneously (sc) injected into the abdominal right flank. Tumor volume was calculated as length × width^2^ × 0.52. For all experiments, tumors were grown up to 100–200 mm^3^ in size (13–14 days) before viral administration. A single viral dose of 1 × 10^7^ plaque forming units (pfu) in 100 μl PBS was injected intravenously (iv) via the tail vein.

### Fluorescence live-animal imaging

Tumor cell growth and viral infection were monitored directly by the RFP expression of tumor cells and GFP expression of Vaccinia virus-infected cells and quantified with the Maestro EX imaging system (CRI, Woburn, MA) using appropriate filters for RFP (tumor; excitation: 503–555 nm, emission: 580 nm cut-in) and GFP (virus; excitation: 445–490 nm, emission: 515 nm cut-in). Images were evaluated and quantified using the Maestro Version 2.10.0 software.

### FACS analysis

For flow cytometric analysis of tumors and exudates, four end-stage PC14PE6 tumor-bearing mice (28 dpim) were sacrificed by CO_2_ inhalation. Effusions were punctured and 400 μl of the exudates were collected. Preparation of tumors were performed as previously described [30]. In both effusion and tumor preparations lysis of erythrocytes using an isotonic ammonium cloride lysis buffer was performed and DNA was digested using 5 MU/ml DNase I.

Blocking of unspecific binding-sites and antibody-labeling using anti-mouse CD45-PECy7 (eBioscience, San Diego, CA, USA) was performed as decribed elsewhere [30]. Immediately before use, dead cells were labeled with propidium iodide (PI) solution.

Labeled cells were subsequently analyzed, using the Accuri C6 Cytometer and FACS analysis software CFlow Version 1.0.227.4 (Accuri Cytometers, Inc. Ann Arbor, MI USA).

### *In vivo* MRI

*In vivo* MRI measurements of tumor-bearing mice were performed at room temperature on a 7 Tesla Bruker Biospec System (Bruker BioSpin GmbH, Reinstetten, Germany) using a 35 mm diameter home-built quadrature birdcage coil. For *in vivo* imaging the animals received inhalation anesthesia (1-2% isoflurane) during the measurement and were placed in a home-built measurement container according to safety regulations.

T_1_ weighted (T_1w_) spin echo experiments were performed at different time points following an injection (iv) of 0.1 mmol/kg body weight Gadopentetate-Dimeglumine (Gd-DTPA, Magnevist, Bayer Schering Pharma AG, Berlin, Germany) of exemplary mock infected animals. Furthermore, for the shown data, a T_2_ weighted (T_2w_) spin echo experiment was performed for anatomical correlation approximately half an hour after injection of the contrast agent.

For n = 4 additional animals (n = 2 mock/ GLV-1h68) multi spin echo (MSE) experiments were performed for the evaluation of tissue T_2_ times (without contrast agent). Data processing of the MRI data was performed in MATLAB (The MathWorks Inc., Natick, USA) using home-written software routines. The chosen MRI sequence parameters are provided in the Additional file 1.

### Protein isolation and hVEGF/mVEGF ELISA of tumor samples

Quantitative evaluation of VEGF concentrations in tumor tissues was performed using a human (Thermo Scientific, EHVEGF) and mouse specific VEGF ELISA kit (abcam, ab100751) according to the manufacturer’s protocol. Tumors were isolated 7 dpi, lysates were prepared as described previously [25]. Absorbance was measured using a Tecan sunrise absorbance reader (Tecan, Crailsheim, Germany). Protein concentrations for each sample were interpolated from a VEGF-specific standard curve.

### RT-PCR of ß-actin

Tissue samples were analyzed for the presence of PC14PE6-RFP cells by RT-PCR. Brains, lungs, and livers of PC14PE6-bearing mice (28 dpim) were homogenized in TRIzol Reagent (Invitrogen) to isolate total RNA. Samples were further treated as described elsewhere [25]. Primer sequence for human ß-actin: (forward: 5*′*-CCT CTC CCA AGT CCA CAC AG-3*′*and reverse: 5*′*- CTG CCT CCA CCC ACT C-3*′*) and for murine ß-actin: (forward: 5*′*-CGT CCA TGC CCT GAG TC- 3*′* and reverse: 5*′*-GCT GCC TCA ACA CCT CAA C-3*′*). PC14PE6-RFP cell lysates were used as positive control for human ß-actin. The PCR reaction was performed in a T-Gradient Thermoblock PCR machine (Biometra, Göttingen, Germany).

### Immunohistochemistry

For histological studies, tumors were excised and snap-frozen in liquid N_2_, followed by fixation in 4% paraformaldehyde/PBS pH 7.4 for 16 h at 4°C. Fixed tumors were rinsed in PBS followed by dehydration in 10% and 30% sucrose/PBS (Carl Roth, Karlsruhe, Germany) and finally embedded in Tissue-Tek® O.C.T. (Sakura Finetek Europe B.V., Alphen aan den Rijn, Netherlands). Tumor samples were sectioned (15 μm) with a cryostat 2800 Frigocut (Leica Microsystems GmbH, Wetzlar, Germany) and stored at −80°C. Antibody-labeling was performed following fixation in ice-cold aceton. The primary antibody was incubated for 1 h. After washing with PBS, sections were labeled for 30 min with the secondary antibody and finally mounted in Mowiol 4–88.

### Whole animal sectioning and H&E staining of tissue sections

Mice were euthanized, perfused with 4% paraformaldehyde, and whole mice were fixed in 4% formalin for 2 days followed by decalcification in 10% formic acid/4% formalin for up to 5 days. Decalcified specimens were dehydrated and embedded in paraffin. Tissue sections (5 μm) were stained by H&E staining.

### Fluorescence microscopy

The fluorescence-labeled preparations were examined using a MZ16 FA Stereo-Fluorescence microscope (Leica) equipped with a digital DC500 CCD camera and the Leica IM1000 4.0 software (1300 × 1030 pixel RGB-color images). Furthermore, a Leica TCS SP2 AOBS confocal laser microscope equipped with argon, helium-neon and UV lasers and the LCS 2.16 software (1024 × 1024 pixel RGB-color images) was used. Digital images were processed with Photoshop 7.0 (Adobe Systems, Mountain View, CA) and merged to generate overlay images.

### Fluorescence intensity measurements and microvessel density

Fluorescence intensity of the CD31-, Ly-6G-, MHCII-labeling as well as the vascular density was measured in 15-μm-thick cryostat sections of control tumors and infected areas of GLV-1h68-colonized tumors on digital images as described earlier [27]. For all experiments the mean value was calculated for nine images (three images each of three different control and GLV-1h68-infected tumors) and presented with standard deviation.

### Antibodies, reagents and treatment of animals

Endothelial cells of blood vessels were labeled with hamster anti-mouse CD31 antibody (Chemicon, International, Temecula, CA). Platelets were labeled with rat anti-mouse CD41 antibody (GeneTex Inc., Irvine, CA). Immune cells were labeled using rat anti-mouse MHCII antibody (B, dendritic cells, monocytes, macrophages) and rat-anti Ly-6G antibody (neutrophils) (eBioscience, San Diego, CA). Vaccinia virus was labeled with rabbit anti-Vaccinia virus (abcam, Cambridge, UK).

The DyLight649-conjugated secondary antibodies (donkey) were obtained from Jackson ImmunoResearch (West Grove, PA).

For the labeling of functional blood vessels in tumors, mice were intravenously injected with 100 μg of biotinylated-*Lycopersicum esculentum* Lectin (Vector Laboratories, Burlingame, CA). Three minutes later, tumors were removed and prepared for histology. Tumor cross-sections (15 μm) were labeled with DyLight649-conjugated streptavidin (Sigma Aldrich, Taufkirchen, Germany) to visualize the Lectin-labeled functional tumor vasculature.

### Statistics

A two-tailed Student’s *t* test was used for statistical analysis. *P* values of < 0.05 were considered statistically significant (*p < 0.05, ** p < 0.01, *** p < 0.001).

## Results

### Subcutaneously implanted PC14PE6-RFP lung adenocarcinomas developed malignant effusions in athymic nude mice

**Figure 1:**
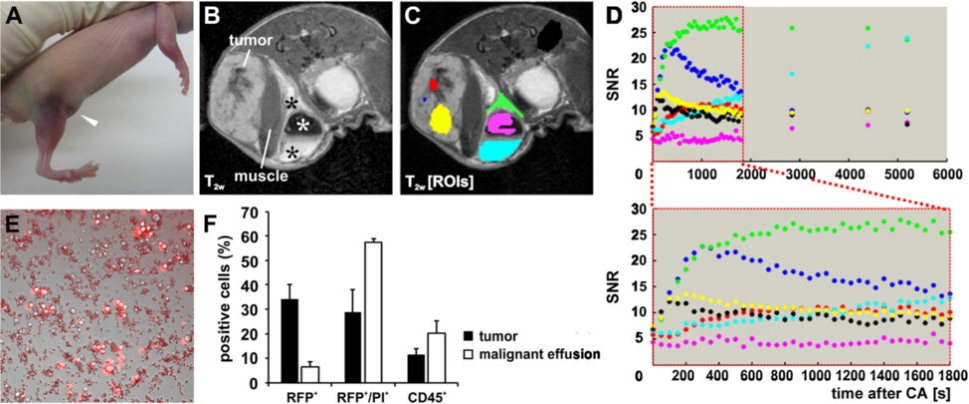
**Generation of subcutaneous PC14PE6-RFP lung adenocarcinomas and tumor-associated ME.** (**A**) Photographic image of a mouse bearing a subcutaneous PC14PE6-RFP tumor on the right flank 14 dpim showing a hematoma around the tumor site and the beginning of exudate accumulation in the groin (arrowhead). (**B-C**) exemplary T_1w_ MRI of a PC14PE6-RFP-bearing mouse 22 dpim revealing tumor-associated effusion in the groin containing fluid (black asterisks, **B**) and solid/semi-solid cellular material (white asterisk, **B**). Using T_1w_ MRI the time-dependent distribution of (iv) injected Gd-DTPA was recorded in different tumor regions and areas of the ME as indicated by regions of interest (ROIs) in (**C**); the corresponding SNR over time of the different ROIs were plotted in (**D**). ROI coloring: red – necrotic tumor region, blue – enlarged blood vessel, yellow – non-necrotic tumor region, green – outer effusion region, pink – solid effusion content, light blue – fluid effusion content, black – control muscle tissue. (**E**) micro-photographic image overlayed with the red fluorescent image of an exudate smear revealing numerous erythrocytes as well as red-fluorescent tumor cells and non-fluorescent cells. (**F**) FACS analysis of PC14PE6-RFP tumors (28 dpim, n = 4) and ME (n = 3); shown are the percentage of RFP-positive tumor cells, propidium iodide (PI)/RFP-positive dead tumor cells, and CD45-positive immune cells in tumors and ME.

Recently, Yano and colleagues reported the formation of MPE of orthotopically generated PC14PE6 lung adenocarcinomas in athymic nude mice [6,7]. In our study, we used the PC14PE6-RFP cells [29] for subcutaneous tumor generation on the flank of athymic nude mice. Despite the different local microenvironment of the lung and the subcutis, we observed the formation of subcutaneous ME 12–14 days post cell implantation (dpim). These peritumoral effusions were visible by eye due to massive subcutaneous blood leakage showing different stages of hematoma formation around the tumor site (Figure 1A). At about 3–4 weeks after implantation all of the tumor-bearing mice developed large and bloody tumor-associated effusions, which consisted of fluid-like as well as semi-solid compartments (up to 800 μl) as depicted by representative T_2w_ images using MRI (Figure 1B).

Exemplarily, the injection of the contrast-agent Gd-DTPA into a tumor-bearing mouse with ME followed by a time course analysis of the contrast agent distribution using T_1w_ MRI revealed a fast and strong accumulation of the contrast agent in well-vascularized tumor areas (blue, Figure 1C, D) as well as in the effusion (green, Figure 1C, D). During the time course the signal to noise ratio (SNR) within the vascularized tumor areas (blue) started to decline 5 min after injection while it further accumulated in the effusion (blue, light blue) reaching a plateau after approximately 15 min.

The Fluorescence-microscopic analysis of the effusion content revealed a large number of erythrocytes, red-fluorescent PC14PE6-RFP tumor cells, as well as non-fluorescent cells, confirming that these effusions are indeed of the malignant type (Figure 1E). Furthermore, FACS analysis of the effusion content showed that most of the cells within the ME were already dead red-fluorescent PC14PE6-RFP tumor cells (57.46%), however, 6.32% of the tumor cells were still alive (Figure 1F). When compared to tumors with only 28.96% dead and 34.32% live tumor cells, the environment of the effusions seems to be cytotoxic. Interestingly, CD45-positive immune cells, which were the other nucleated cell component of effusions, were enriched in ME (20.26%) when compared to tumors (11.63%) indicating an inflammatory environment of ME.

Taken together, the results showed that PC14PE6 tumor-associated ME can be established in the flank area of mice indicating that the local microenvironment of the lung is not essential for ME formation.

### PC14PE6-RFP tumors exhibited enlarged CD31-positive blood vessels containing clusters of tumor cells

**Figure 2:**
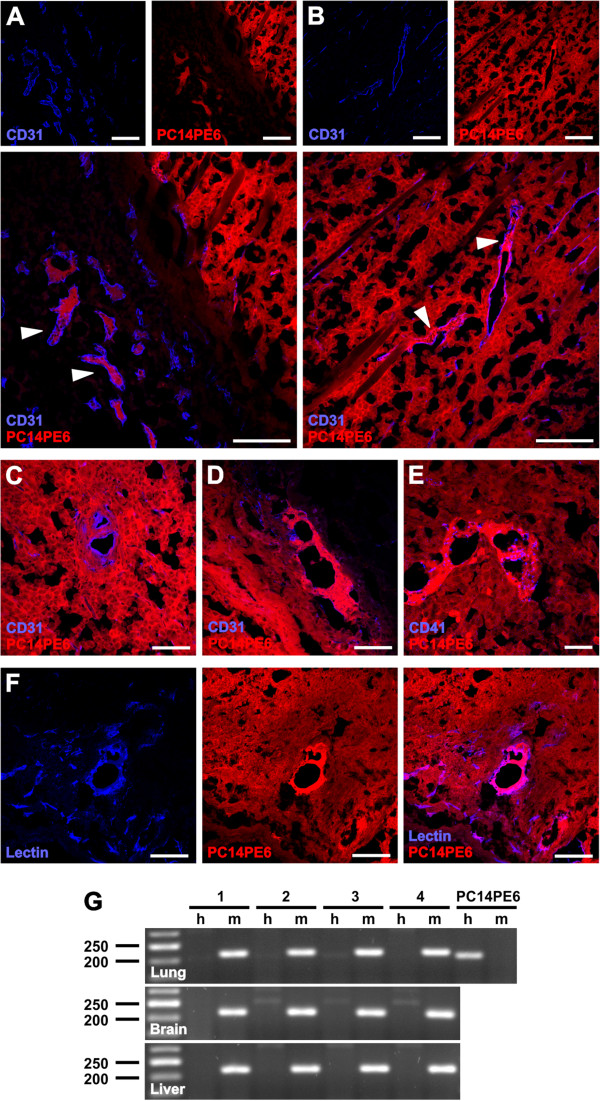
**Enlarged tumor cell-containing CD31-positive blood vessels (TCCBVs) in PC14PE6-RFP tumors.** (**A-F**) Confocal microscopic images of 15 μm-thick PC14PE6-RFP tumor sections 21 dpim showed enlarged CD31-positive blood vessels (blue) containing RFP-expressing tumor cells (red, arrowheads) in peritumoral regions (**A**) and tumor border areas (**B**). In tumor sections enlarged CD31-positive TCCBVs appear in various morphological formations resembling glomeruloid bodies (**C**) or garland-like vessels with intussusceptive vascular growth (**D**). Functionality of enlarged TCCBVs was confirmed by co-localization of CD41-positive platelets with garland-like TCCBVs (**E**) and Lectin-labeling of TCCBVs upon systemic injection of Lectin into PC14PE6-RFP-bearing mice. Both microscopic images indicate the connection of TCCBVs to the blood circulation. (**G**) RT-PCR analysis of lung, brain and liver homogenates of end-stage PC14PE6-RFP-bearing mice (28 dpim) using human- (h, 205 bp) and mouse-specific (m, 216 bp) ß-actin primers (n = 4). Human PC14PE6-RFP cells were used as a positive control for human ß-actin and as a negative control for mouse ß-actin. All images are representative examples. Scale bars represent 300 μm (**A**, **B**, **F**), 75 μm (**C**, **D**), and 40 μm (**E**).

Orthotopically established PC14PE6-RFP lung adenocarcinomas contained a significant proportion of blood vessels with a large lumen and some of them with transluminal bridges of endothelial cell branches, which are hallmarks of non-sprouting angiogenesis [31]. In this study, we performed confocal-laser scanning microscopy of whole tumor cross-sections of subcutaneously established PC14PE6-RFP tumors and detected enlarged CD31-positive vessels. However, within these enlarged CD31-positive blood vessels we observed clusters of RFP-positive PC14PE6 tumor cells, which either lined the inner surface of the vessels or filled the lumen of the vessels (Figure 2A, B). We termed these *tumor cell-containing CD31*^*+*^*blood vessels* as TCCBVs. Enlarged CD31^+^ TCCBVs were localized mainly in outer tumor regions or peritumoral areas and showed a heterogeneous morphology – reaching from complex vascular structures such as glomeruloid bodies to structures with variable intussusceptive vascular growth (Figure 2C, D). Labeling of CD41-positive platelets revealed a co-localization of TCCBVs and platelets indicating the connection to the circulation (Figure 2E). To further analyze the functionality of CD31^+^ TCCBVs, we injected (iv) biotinylated *Lycopersicon esculentum* Lectin to only label blood vessels which are actively connected to the blood stream. We could identify Lectin-labeled TCCBVs indicating that these special blood vessels were still functional (Figure 2F).

Since PC14PE6-RFP tumors were described to be highly metastatic and easily form brain metastases [31], the intra-vascular PC14PE6-RFP cells may be actively migrating metastatic tumor cells. Therefore, we analyzed different organs of late-stage PC14PE6-RFP tumor-bearing mice (28 dpim) for local metastases using human-specific ß-actin primers as target for RT-PCR analysis. Interestingly, neither brains nor lungs or livers of the analyzed mice were positively tested for human PC14PE6-RFP tumor cells (Figure 2G). Thus, we assume that TCCBVs are statically associated with the tumor vasculature of PC14PE6-RFP lung adenocarcinomas rather than are being snapshots of metastatic seeding.

### rVACV colonization of PC14PE6-RFP tumors retained both tumor growth and ME formation

**Figure 3:**
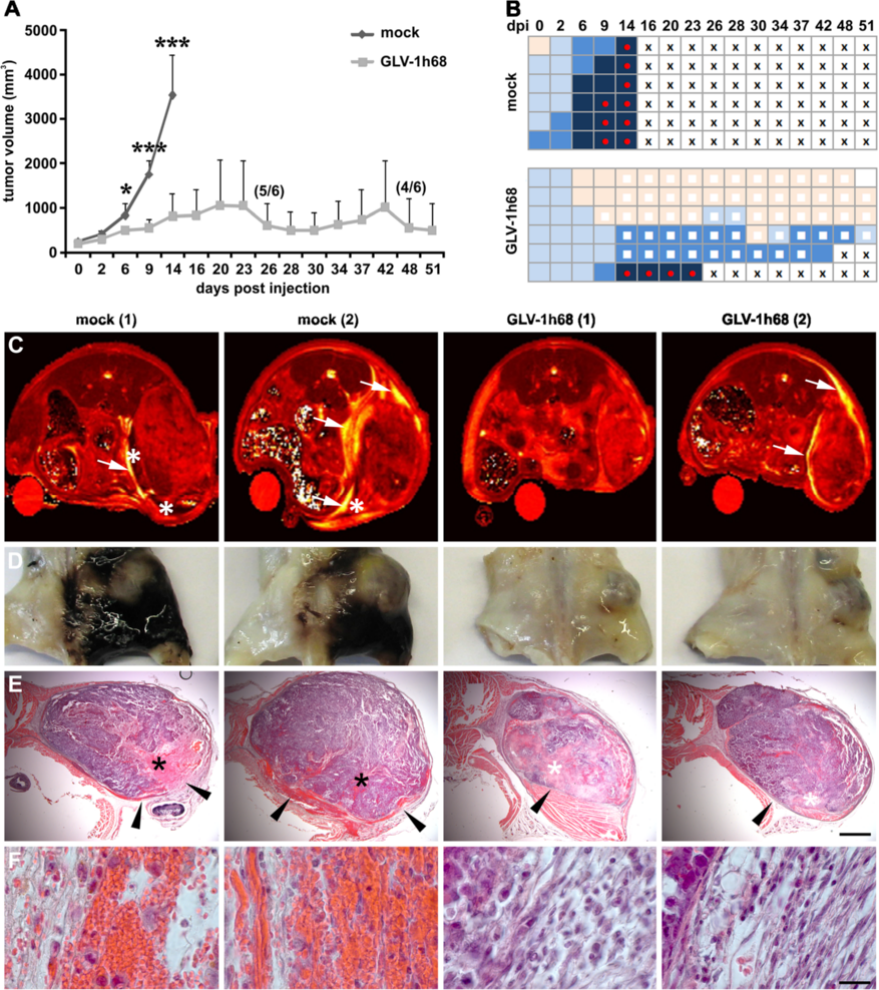
**Therapy of ME in PC14PE6-RFP tumor-bearing mice upon systemic injection of GLV-1h68.** (**A-B**) PC14PE6-RFP tumor-bearing mice were either mock-infected or treated with 1 × 10^7^ pfu GLV-1h68 (iv) 14 dpim (n = 6). (**A**) PC14PE6-RFP tumor growth was monitored by measuring the tumor volume revealing a continuous tumor growth in the mock-infected group and a significantly retarded growth rate in the GLV-1h68-treated group; all mice of the mock-infected group were euthanized due to tumor burden and ME formation 14 dpi; in the GLV-1h68-treated group two mice were euthanized due to ME formation (23, 42 dpi) before end of the study. Shown are the mean values +/− standard deviations. (**B**) same mice as shown in A were individually monitored for ME formation during the study; ME formation was evaluated by skin color of the tumor area (hematoma) and exudate accumulation in the groin; skin color index indicating increased hematoma: skin-coloured – light blue – middle blue – dark blue; red dot: palpable cyst in the groin (exudate accumulation), white square: area of tumor necrosis, cross: euthanization. (**C**) *in vivo* MR T_2_ maps of two mock- and two GLV-1h68-infected PC14PE6-RFP tumor-bearing mice 7 dpi. Tumor-associated effusions (arrow) with accumulation of solid/semi-solid cellular components (asterisk) are indicated in T_2_ images. The T_2_ maps are scaled from 0 to 200 ms. (**D**) the corresponding photographic images of the paraformaldehyde-fixed and decalcified mouse abdomen demonstrated the extended hematoma in the peritumoral area of mock-infected mice; in GLV-1h68-treated mice no peritumoral hematoma was detected. (**E, F**) H&E staining of the corresponding whole abdominal mouse sections revealed erythrocytes-containing blood lakes (black asterisks, E) in mock-treated and necrotic tumor areas (white asterisk, E) in GLV-1h68-treated mice. Different histopathology of the tumor margin of mock- and GLV-1h68-treated tumors (arrowheads, E) is shown with higher magnification in (**F**). All images are representative examples. Scale bars represent 2 mm (**E**) 20 μm (**F**).

To test efficacy of an oncolytic virotherapy of PC14PE6-RFP lung adenocarcinomas using the recombinant Vaccinia virus GLV-1h68, we monitored tumor growth after systemic treatment. As shown in Figure 3A rVACV significantly reduced overall tumor growth and prolonged survival time, however, with high interindividual variation in the therapeutic effectiveness. For example, the first rVACV-treated mouse had to be euthanized due to tumor burden already 23 dpi, compared to another rVACV-treated mouse with complete tumor disappearance 51 dpi. At the same time, we monitored the formation of ME in all rVACV-treated and untreated tumor-bearing animals. Most of the animals already developed ME initially shown as hemorrhagic areas around the tumor site at the onset of the treatment. Interestingly, in approximately 50% of the rVACV-treated mice the effusions completely disappeared, in the other cases ME formation was at least highly attenuated (Figure 3B).

To determine the anatomical context of ME, we scanned untreated and virus-treated animals first by *in vivo* MRI (Figure 3C) followed by whole mouse sectioning (Figure 3D-F). In untreated PC14PE6-RFP tumor-bearing mice ME was visualized already 7 dpi by MRI (Figure 3C). These animals showed highly necrotic tumors surrounded by enlarged effusions. In contrast, rVACV-treated tumors showed highly attenuated ME. Histological analysis of PC14PE6-RFP tumor-associated ME revealed erythrocyte-containing blood lakes with highest frequency at the tumor rim in untreated tumors (Figure 3E, F). Moreover, these tumors did not show defined tumor borders and blood leaks into the surrounding tissue/cavity. In GLV-1h68-treated tumors defined tumor border regions and extended necrotic tumor areas were observed.

In summary, oncolytic therapy using rVACV proved to be an effective treatment modality for both growth control of lung adenocarcinomas and therapy of ME.

### rVACV colonization of PC14PE6-RFP tumors activates the tumor endothelium

**Figure 4:**
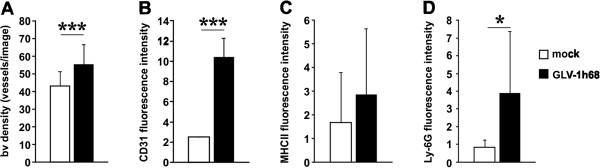
**VACV infection-induced activation of the tumor vasculature.** (**A-D**) PC14PE6-RFP tumor-bearing mice were either mock-infected or treated with 1 × 10^7^ pfu GLV-1h68 (iv) 14 dpim (n = 3). Seven dpi tumors were harvested and histological tissue sections were performed. The blood vessel density (**A**) was determined by counting the vessels/image in both groups and the mean fluorescence intensity of endothelial-associated CD31 was shown in (**B**). The content of either antigen-presenting MHCII-positive cells (monocytes, macrophages, dendritic cells, and B cells) (**C**) or Ly-6G-positive neutrophils (**D**) in mock- and GLV-1h68-treated tumors was determined by quantification of the mean fluorescence intensity of the antibody-labeled tumor sections.

Since rVACV inhibits the formation of ME in PC14PE6-RFP tumor-bearing mice, we investigated the direct effect of rVACV infection on the tumor vasculature. Contrary to our hypothesis that rVACV treatment may reduce vascular density and thus prevents ME formation, we detected a significant increase in the CD31-positive vascular density of rVACV-treated tumors (Figure 4A). Furthermore, we identified an increased CD31-fluorescence intensity on endothelial cells in rVACV-treated tumors. This indicates an enhanced accumulation of leukocytes, since CD31 is involved in leukocyte trafficking to sites of inflammation (Figure 4B). Indeed, we found increased accumulations of macrophages and neutrophils in rVACV-treated tumors (Figure 4C, D).

Altogether, rVACV treatment of PC14PE6-RFP tumors led to inflammation-mediated activation of the endothelium and did not lead to a direct destruction of the tumor vasculature.

### rVACV colonization of PC14PE6-RFP tumors led to a decrease in the intratumoral human VEGF (hVEGF) content and to infected TCCBVs

**Figure 5:**
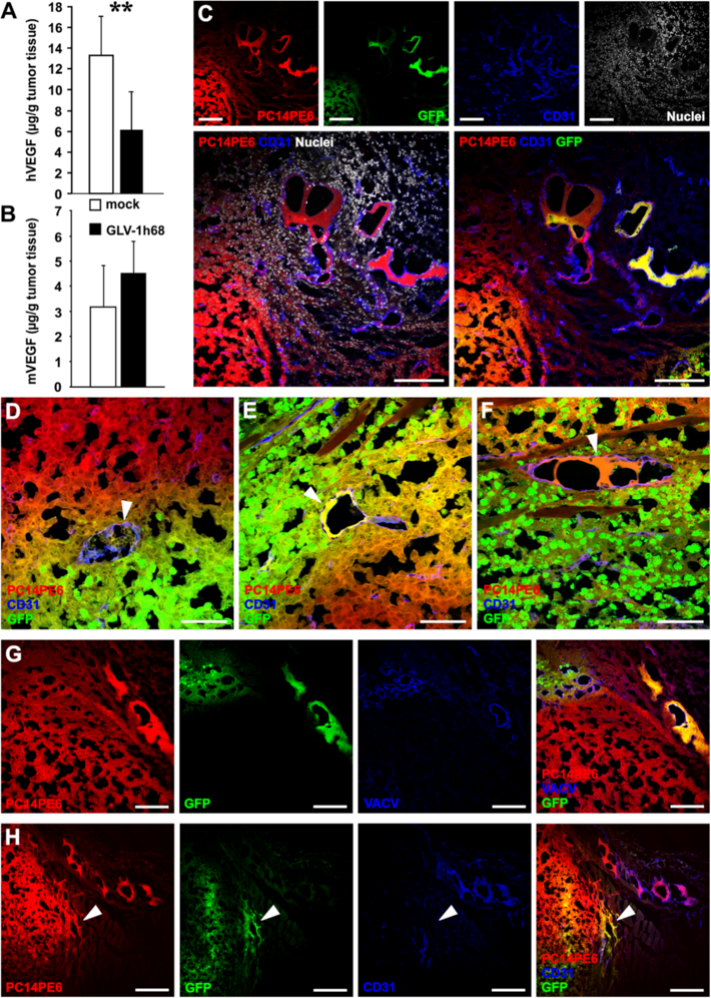
**VACV treatment decreased tumor cell-produced hVEGF and infected TCCBVs in PC14PE6-RFP tumors.** (**A, B**) PC14PE6-RFP tumor-bearing mice were either mock-infected or treated with 1 × 10^7^ pfu GLV-1h68 (iv) 14 dpim. Tumor homogenates of mock- and GLV-1h68-treated tumors (7dpi, n = 6) were used for ELISA of human VEGF (**A**) and murine VEGF (**B**). Shown are the mean values +/− standard deviations. (**C-F**) Histological analysis of GLV-1h68-infected PC14PE6-RFP tumor sections 7 dpi revealed a GLV-1h68-infection of TCCBVs; PC14PE6-RFP tumor cells (red), GLV-1h68-infected cells express GFP (green), blood vessels were labeled with the CD31 antibody (blue), nuclei were labeled with Hoechst 33324 (white). All the different morphological formations of TCCBVs such as glomeruloid bodies (**D**) and garland-like vessels (**E, F**) were GFP-positive. (**G**) anti-VACV antibody labeling (blue) of tumor sections revealed VACV-positive labeling in GFP-positive areas. (**H**) Histological analysis of GLV-1h68-infected PC14PE6-RFP tumor sections revealed GLV-1h68-infection of invasive PC14PE6-RFP tumor cells at the tumor margin (arrowhead); PC14PE6-RFP tumor cells (red), GLV-1h68-infected cells (green), CD31-positive blood vessels (blue). All images are representative examples. Scale bars represent 300 μm (C, G), 75 μm (D-F), and 150 μm (H).

Previously, it was shown that tumor-cell derived VEGF is one of the most important factors contributing to ME formation in PC14PE6-RFP tumors [6]. Therefore, we analyzed the effect of rVACV treatment on the levels of tumor cell-derived hVEGF (7 dpi). We found significant decreased levels of tumor cell-derived hVEGF in rVACV-treated tumors (Figure 5A). In contrast, the murine VEGF deriving mostly from host immune cells was not affected (Figure 5B), indicating the tumor cell specificity of rVACV infection.

Investigation of rVACV-treated tumors (7dpi) revealed that tumor cells located in CD31-positive TCCBVs did express GFP, a direct visual marker for viral infection (Figure 5C). Interestingly, different morphologies of TCCBVs such as glomeruloid bodies and vessels with intussusceptive vascular growth were infected by rVACV (Figure 5D-F). Moreover, specific anti-VACV antibody labeling of tumor sections confirmed viral infection of TCCBVs by positively labeling of GFP-positive tumor areas (Figure 5G). In addition to the VEGF over-expression the invasive potential of the tumor cells seems to be critical for ME formation [6]. The investigation of the tumor border region in rVACV-treated tumors revealed GLV-1h68-infected PC14PE6-RFP tumor cells, thereby directly preventing tumor cell invasion of the peritumoral tissue (Figure 5H).

In summary, viral colonization of PC14PE6-RFP tumors decreased tumor cell-derived hVEGF levels, directly infected TCCBV’s, and inhibited tumor cell invasion of the peritumoral tissue, thereby rVACV potentially prevented the formation of ME.

### rVACV encoding for GLAF-1 significantly enhanced both therapy of lung adenocarcinomas and ME

**Figure 6:**
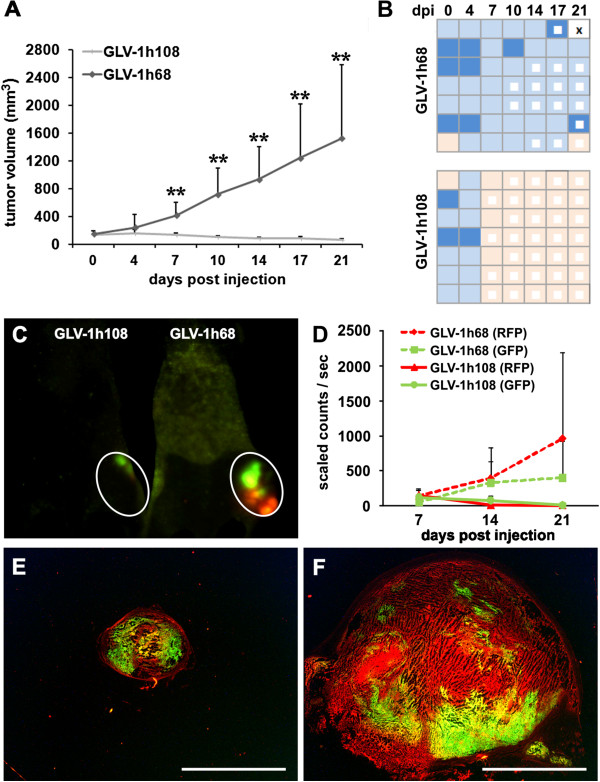
**Enhanced therapeutic efficacy in tumor xenografts with ME using an anti-VEGF scAb-encoding VACV.** (**A-F**) PC14PE6-RFP tumor-bearing mice were either iv infected with 1 × 10^7^ pfu GLV-1h68 or 1 x10^7^ GLV-1h108 14 dpim (n = 7). (**A**) PC14PE6-RFP tumor growth was monitored over 21 days pi by measuring the tumor volume revealing a continuous tumor growth in the GLV-1h68-infected group and a significant tumor regression in the GLV-1h108 treated group. Shown are the mean values +/− standard deviations. (**B**) same mice as shown in A were individually monitored for ME formation during the study; ME formation was evaluated by skin color of the tumor area (hematoma); skin color index indicating increased hematoma: skin-coloured – light blue – middle blue; white square: area of tumor necrosis, cross: euthanization. (**C, D**) real-time fluorescence imaging of tumor growth (RFP) and viral infection (GFP) during growth curve analysis at day 7, 14, and 21 pi using the Maestro EX imaging system. (**C**) representative fluorescence image of GLV-1h68- and GLV-1h108-infected PC14PE6-RFP-tumor-bearing mice 14 dpi. (**E,F**) representative microscopic images of 15 μm-thick PC14PE6-RFP tumor sections 21 dpi either infected with GLV-1h108 (**E**) or GLV-1h68 (**F**); PC14PE6-RFP tumor cells (red), viral infected GFP-expressing cells (green). All images are representative examples. Scale bars represent 5 mm (**E**, **F**).

Recently, we showed that the anti-VEGF single-chain antibody GLAF-1 encoded by oncolytic Vaccinia virus (GLV-1h108) significantly enhanced anti-tumor therapy in different tumor models [28]. Here, we investigated whether the increased capture of the intratumoral overexpressed hVEGF by viral encoded GLAF-1 may improve therapy of ME. Monitoring of tumor growth after systemic injection of both viral strains (GLV-1h68 and GLV-1h108) revealed significantly increased tumor regression in the GLV-1h108-treated in comparison to the GLV-1h68-treated group (Figure 6A). Moreover, the initially observed tumor-associated hematoma disappeared in all the mice of the GLV-1h108-treated group at 7 dpi (Figure 6B). Fluorescence-imaging of PC14PE6-RFP tumor growth (RFP) and GLV-1h68 or GLV-1h108 infection (GFP) revealed that in GLV-1h68-infected animals both the RFP and the GFP signal increased during the 21-day observation period (Figure 6C, D). However, the RFP signal outgrows the GFP signal at later time points indicating that the viral infection progressed slower than tumor growth *per se*. In contrast, in GLV-1h108-injected animals both the RFP and the GFP signal simultaneously declined indicating the progress of virus-dependent tumor growth elimination. Whole tumor sections 21 dpi confirmed the imaging data and demonstrated that the GLV-1h108 virus spreads throughout most of the tumor tissue, whereas GLV-1h68 showed a patchy viral distribution pattern with large areas of active tumor growth (Figure 6E, F).

Taken together, these data indicate that both tumor growths of PC14PE6-RFP lung adenocarcinomas as well as formation of ME was efficiently controlled by treatment with oncolytic rVACV GLV-1h108 encoding for the scAb against VEGF in mice.

## Discussion

Late-stage cancer is often accompanied by the formation of ME, which are often resistant to conventional anti-neoplastic therapy and thus represent a major challenge in oncology. In this study, we showed that virotherapy using Vaccinia virus as an oncolytic agent and as a drug-delivering vehicle may become a promising therapeutic strategy for the treatment of lung adenocarcinoma-associated ME. So far, using a Measles virus strain as oncolytic agent, only Iankov et al. [32,33] reported therapeutic effectiveness of oncolytic virotherapy in a malignant pleural effusion breast cancer model. In addition, we showed for the first time that oncolytic virotherapy decreases tumor-derived hVEGF levels, leads to viral infection of TCCBVs, and inhibits tumor cell invasion of the peritumoral tissue, thereby preventing formation of ME. Interestingly, VACV treatment did not lead to destruction of the tumor vasculature. Therefore, therapy of ME was not dependent on virus-mediated vessel destruction. Finally, the use of the recombinant Vaccinia virus strain GLV-1h108 encoding for a scAb against VEGF significantly enhanced the oncolytic mono-therapy.

In the present study, we found that the formation of ME in the PC14PE6 lung adenocarcinoma model was independent of the local pleural microenvironment. After generation of subcutaneous lung xenografts on the flank of mice we observed tumor-associated ME, elevated intratumoral VEGF levels, enlarged blood vessels and tumor cells invading the peritumoral space in 100% of the mice. These findings are in accordance with previous findings for the orthotopic model [6]. Despite of the “artificial” character of the subcutaneous tumor ME model several aspects contribute to the direct relevance of this model to MPE pathogenesis in cancer patients. First of all, in the subcutaneous tumor-ME model as well as in patients ME are the results of fluid accumulation in body cavities due to increased fluid formation and/or reduced drainage. Second, in both situations VEGF and the invasive potential of tumor cells are main contributors to increased fluid accumulation and leakage in nearby body cavities. Finally, the main differences of MPE in patients and ME in the subcutaneous tumor-ME model are the location of the body cavities where the exudates accumulate – the pleura versus the “cavity” between the subcutis and the peritoneum.

In contrast to orthotopic MPE, subcutaneous ME at the flank are non-invasively detectable due to hematoma formation in the skin and/or exudates accumulation in the groin. Thus, for monitoring of ME there is no need to sacrifice the mice or need for special instrumentation such as MRI scanners. Therefore, we propose that this subcutaneous model of ME may be an easier-to-handle model with comparable characteristics of the published orthotopic model and is especially suited for the screening of therapeutic interventions for tumor-associated ME.

VEGF is a well established mediator of vascular permeability and can be found in significant higher concentrations in effusions of malignant compared to non-malignant origin [34-36]. Several groups have already shown that the anti-VEGF-based therapy using Bevacizumab profoundly counteracts effusion formation in patients with various solid tumors [37,38]. Therefore, VEGF seems to be one of the important factors contributing to local hyperpermeability leading to tumor-associated ME. Several cell types such as leukocytes, mesothelial cells, and cancer cells may serve as a potential source for vasoactive VEGF. By the use of human tumor cells in a murine host context, we were able to differentiate between the hVEGF produced by tumor cells and the mVEGF produced by host cells such as leukocytes. As oncolytic virotherapy specifically reduces hVEGF and not mVEGF accompanied by resolution of ME, the results indicate that the most important sources for VEGF were the tumor cells itself. Furthermore, the results demonstrated that mVEGF-producing cells were not efficiently infected and lysed by oncolytic virotherapy using rVACV. The important role of tumor cell-derived VEGF in this model of ME was further demonstrated by the use of a rVACV strain encoding for therapeutic antibodies against VEGF (GLAF-1) in the direct local proximity to VEGF-producing cancer cells, which significantly enhances the therapeutic effect on ME formation.

Moreover, the invasive potential of PC14PE6 tumor cells seems to be critical for the formation of ME [6]. This is due to the disruption of the tumor capsule, which facilitates the leakage of interstitial fluid into the peritumoral tissue or body cavities. Interestingly, oncolytic virotherapy seems to alter the invasive potential of PC14PE6 tumor cells hence VACV-treated tumors revealed defined tumor capsules.

Yano and colleagues established the orthotopic PC14PE6 malignant effusion lung adenocarcinoma model and reported in elegant publications the presence of an abnormal tumor vasculature in PC14PE6 tumors [6,31]. Nowadays, it is widely accepted that the abnormalities of the tumor vasculature contribute significantly to the disturbed microenvironment of solid tumors. This may finally result in a highly disturbed blood flow which does not follow a constant, unidirectional path in tumors resulting in local hyperpermeability or zones of ischaemia [39]. Furthermore, Yano et al. reported that a large proportion of blood vessels exhibited large lumina with transluminal bridges of endothelial processes [31], which are hallmarks of non-sprouting angiogenesis [40]. In accordance, we observed the existence of enlarged blood vessels with transluminal bridges in PC14PE6 tumors. Moreover, by the use of RFP-expressing PC14PE6 tumor cells, mouse-specific anti-CD31 antibodies, and Vaccinia virus infection, we were able to identify tumor cells within these enlarged vessels (TCCBVs) forming transluminal bridges. Our data furthermore indicated that the invasive tumor cells did not contribute to distant metastases formation, however, we do not know whether TCCBVs were the result of the abnormal high intratumoral VEGF level or other microenvironmental abnormalities. However, a study from Dvorak et al. [41] indicated that VEGF is synthesized by tumor cells *in vivo* and accumulates in nearby blood vessels. Furthermore, it was reported that the chemotactic action of VEGF enables tumor cell-trans-endothelial migration [42] and tumor cells may more easily penetrate a retracted endothelial monolayer caused by VEGF than a tightly arranged monolayer. This suggests that the vascular permeability activity of VEGF contributes to an “offensive ability” of the tumor cells, allowing them to penetrate blood vessels [43]. Taken together, we speculate that TCCBVs may be the result of an abnormal high VEGF accumulation in blood vessels of tumors followed by migration and invasion of the endothelium by tumor cells.

Besides the strong effect of the rVACV-mediated inhibition of VEGF on ME formation other mechanisms such as the VACV-induced inflammatory host response may contribute to the resolution of ME. Reports of using bacterial cell-derived immunomodulatory preperations such as OK-432 as successful therapeutics in patients with MPE indicate the importance of the host immune response to therapy outcome [44-46]. In addition, recently published data showed that an immunostimulatory transgene encoded by oncolytic measles virus significantly enhanced the anti-tumor effect in xenograft models of metastatic cancer, including malignant pleural effusion model. To elucidate whether the VACV-mediated host immune response contributes to the resolution of ME is the focus of ongoing research.

## Conclusions

In conclusion, this study showed that oncolytic virotherapy of lung adenocarcinomas resolved and prevented formation of ME mainly via decreasing tumoral VEGF production, infection of TCCBVs and inhibition of invasion of the peritumoral space. The significantly enhanced therapeutic efficacy using rVACV encoding for scAb against VEGF further highlights the promising application of recombinant oncolytic Vaccinia viruses encoding for specific immuno-therapeutic antibodies enabling targeted therapy.

## Abbreviations

dpi: Days post infection; dpim: Days post implantation; ELISA: Enzyme-linked immunosorbent assay; FACS: Fluorescence-activated cell sorting; FBS: Fetal bovine serum; Gd-DTPA: Gadopentetate-Dimeglumine; GFP: Green fluorescent protein; iv: Intravenous; ME: Malignant effusion; MPE: Malignant pleural effusion; MRI: Magnetic resonance imaging; MSE: Multi spin echo; pfu: Plaque forming unit; PI: Propidium iodide; RFP: Red fluorescent protein; rVACV: Recombinant Vaccinia virus strain; sc: Subcutaneous; scAb: Single-chain antibody; SNR: Signal to noise ratio; T1w: T1 weighted; T2w: T2 weighted; TCCBV: Tumor cell-containg blood vessel; VEGF: Vascular endothelial growth factor.

## Competing interests

SW, EH, UD, and CS received a postdoctoral fellowship, TCBL, MA, and PG received a graduate fellowship from a service grant provided by Genelux Corporation awarded to the University of Wuerzburg, Germany. AF, IG and AAS are salaried employees of Genelux Corporation and have personal financial interests in Genelux Corporation. The funders had no role in study design, data collection and analysis or decision to publish.

This work was supported by a research grant and a service grant from Genelux Corporation (R&D facility in San Diego, CA, USA) awarded to the University of Wuerzburg, Germany.

## Authors’ contributions

SW and EH conceived and designed the study, performed experiments, analyzed the data. SW wrote the manuscript. TCBL, UD, CS, MA, PG, CK performed experiments and analyzed the data. AF and IG approved the final manuscript version. PMJ provided MRI equipment and approved the final version of the manuscript. AAS conceived the study and helped to draft and edit the manuscript. All authors read and approved the final manuscript.

## Supplementary Material

Additional file 1 **Parameters for the different *in vivo *MRI experiments.** Click here for file
