# Supplementary material for: Treatment of malignant effusion by oncolytic virotherapy in an experimental subcutaneous xenograft model of lung cancer
Source: J Transl Med. 2013 May 1;11:106. doi: 10.1186/1479-5876-11-106 (PMC3646671; doi:10.1186/1479-5876-11-106)
Supplement: Additional file 1 — Parameters for the different in vivo MRI experiments. [file 1479-5876-11-106-S1.docx]

**Additional information 1: Parameters for the different *in vivo* MRI experiments.**

| Sequence | Fig | TE [ms] | TR [ms] | TF | NE | MTX | FOV |
| --- | --- | --- | --- | --- | --- | --- | --- |
| T_1w_ (SE) | 1 | 5.9 | 400 | 1 | 1 | 125 x 125 | 25 x 25 |
| T_2w_ (TSE) | 1 | 30 | 7000 | 10 | 1 | 130 x 130 | 25 x 25 |
| T_2_ (MSE) | 3 | 6* | 5000 | 1 | 20 | 125 x 150 | 25 x 30 |

Chosen acronyms: SE = spin echo, TSE = turbo spin echo, MSE = multi spin echo, Fig = figure, TE = echo time, TR = repetition time, TF = turbo factor for TSE experiments, NE = number of echoes for MSE experiments, MTX = imaging matrix, FOV = field-of-view. All sequences were 2D multi slice sequences with 20 slices and a slice thickness of 1 mm. *Regarding the MSE experiments, TE stands for the echo time of the initial echo and the time between the subsequent echoes.
